# Optical and Thermomechanical Properties of Doped Polyfunctional Acrylate Copolymers

**DOI:** 10.3390/polym10030337

**Published:** 2018-03-19

**Authors:** Thomas Hanemann, Kirsten Honnef

**Affiliations:** 1 Karlsruhe Institute of Technology, Institute of Applied Materials, Hermann-von Helmholtz-Platz 1, D-76344 Eggenstein-Leopoldshafen, Germany; 2 Laboratory for Materials Processing, Institute for Microsystems Technology, Albert-Ludwigs-University of Freiburg, Georges-Koehler-Allee 102, D-79110 Freiburg, Germany; honnef@imtek.de

**Keywords:** refractive index tailoring, optical transmittance, polymer waveguides, photoinitiated curing, photopolymerization, UV-curing

## Abstract

Three different polyfunctional acrylate monomers—trimethylolpropantriacrylate (TMPTA), pentaerythritol triacrylate (PETA) and di(trimethylolpropane) tetraacrylate (DTTA)—have been used as comonomers in combination with a reactive resin consisting of poly(methylmethacrylate), dissolved in its monomer methylmethacrylate. Phenanthrene has been added to form a guest–host system. The level of phenanthrene present may be adjusted to tailor the refractive index in the system. Prior to curing, the shear rate and temperature-dependent viscosity as a function of the composition were measured. It could be demonstrated that, with respect to different shaping methods, a tailor-made flow behaviour can be adjusted. After thermally-induced polymerization, the resulting optical (refractive index, optical transmittance) and thermomechanical (glass transition behavior, Vickers hardness) properties were characterized. A significant refractive index increase—up to a value close to 1.56 (@589 nm)—under the retention of good optical transmittance was able to be obtained. In addition, the photopolymerization behaviour was investigated to overcome the undesirable oxygen inhibition effect during the light-induced radical polymerization of acrylates. The level of acrylate units in the copolymer can compensate for the plasticizing effect of the dopant phenanthrene, enabling higher concentrations of the dopant in the guest–host system and therefore larger refractive index values suitable for polymer waveguide fabrication.

## 1. Introduction

Due to the rapid increase in data traffic both in the private as well as in the business sector, which is being accelerated by the expanding internet, classical copper-cable-based wideband lines are reaching their limits. Therefore optical data transmission is increasingly important due to higher data transmission rate, lower damping and improved security. Beyond the application of the established fused silica cables in the fiber-optic network, polymer optical fibers are gaining more and more importance for short distance data transmission, e.g., in the automotive sector, due to their low weight and higher bending flexibility. In addition, the further development of customized wearables containing sensors for, e.g., health monitoring, requires the use of flexible polymer waveguides with tailored characteristics [1,2,3,4,5,6,7]. In fact, only very few polymers with acceptable optical properties are available commercially. The most important of these for technical applications are poly(methylmethacrylate) (PMMA), poly(methylmethacrylimide) (PMMI), poly(carbonate) (PC) and the cycloolefinic copolymers (COP, COC). Each of these possess a fixed set of physical attributes and can be replicated with established methods such as injection molding and related techniques for the fabrication of optical components, e.g., lenses or data storage devices such as CD/DVD/Blu Ray disks.

With respect to the fabrication of customized devices, such as polymer waveguides with passive or active optical properties, new materials with tailored features including refractive index and optical transmittance have to be developed. In addition, these new materials have to be adapted to the surroundings, such as attached light sources and detectors, as well as to accommodate geometric device characteristics. Furthermore, thermomechanical properties such as glass transition temperature (*T*_g_), which is relevant for the device maximum operation temperature, must be adapted. The Vickers hardness can be used as a measure of surface stability. All material properties must also be adjusted to meet the requirements of the individual replication methods, such as the various melt processing techniques like hot embossing, injection molding or derived methods [8,9]. Ambient temperature processing methods—the shaping of curable resins, which often consist of polymers dissolved in their monomers—and the subsequent thermal or UV-curing—light induced polymerization or photo-polymerization—are gaining more and more importance. Examples that utilize photopolymerization are inkjet or flexo/offset printing, reaction molding, wafer-level microreplication [10], nanoimprint lithography and stereolithography (SLA). Quite recently, polymer optical waveguides and optical scattering layers for OLEDs (Organic Light Emitting Diodes) from customized resins and inkjetting with subsequent UV-curing have been reported [11,12,13].

In the last couple of years, different attempts at adjusting the optical and thermomechanical properties of polymers have been undertaken. Initially, highly refractive ceramic nanoparticles like alumina, zirconia, or titania were dispersed in reactive resins to increase the refractive index. Since changes in the refractive index correlate with the nanofiller load, only small changes could be achieved because of significant increases in resin viscosity, which hindered any shaping. The loss of optical transmittance due to particle agglomeration and resulting light scattering also represented limitations [3,14,15,16].

Since the refractive index correlates with the number of polarizable units—e.g., an aromatic system in an organic molecule, as described by the Clausius–Mosotti (Lorentz–Lorenz) equation—copolymers with electron-rich moieties in the main or side chain have been synthesized [1]. Polymer purification to achieve optical quality is time consuming and represents a further limitation. Rapid material screening is therefore impossible. The formation of guest–host systems with a suitable electron rich molecule dissolved in a polymer matrix provides a more suitable approach [17,18,19]. Earlier work investigated a series of different electron-rich molecules with respect to their solubility in different photocurable resins and their impact on optical and thermomechanical properties [19]. The largest effects due to their enhanced solubility in MMA/PMMA-based polymer matrices could be observed in case of phenanthrene and benzochinoline [19]. Some of the molecules caused a yellowing of the mixtures, which cannot be accepted for optical applications. Due to pricing (benzochinoline is five times more expensive), phenanthrene was selected for further comprehensive investigation. Therefore, the use of phenanthrene is the best compromise between pricing, solubility in the used monomer mixtures, refractive index enhancement and lowest coloring (yellowing) in the visible range.

As a drawback, the presence of small dopant molecules causes a pronounced reduction of the glass transition temperature range due to plasticizing. This is observed for different unsaturated polyester–styrene and methyl methacrylate–poly(methyl methacrylate) (MMA/PMMA)-based polymer matrixes [19]. The addition of a polyfunctional monomer as a comonomer to increase the crosslinking density has the potential to partially compensate the plasticizing effect. This has been shown for doped polymerized unsaturated polyester/styrene [17] and poly(methyl methacrylate-co-1,3-butyl dimethacrylate) [20]. In contrast, the use of ethyl glycol dimethyl methacrylate (EGDMA) as a comonomer did not reduce the softening behaviour of the cured resin [20].

Polyfunctional (meth)acrylates are widely used in orthodontics as one component of the applied curable resins to enable good mechanical stability [21]. More current work describes complex, highly crosslinked, transparent trimethylamine acrylates and methacrylates with improved thermomechanical stability. These were obtained from monomers with three (meth)acrylate moieties in one molecule targeting scratch-resistant, UV-curable coatings [22]. Furthermore, polyfunctional acrylates are part of modern UV-curable inks used in flexo- or inkjet printing to enhance e.g., the curing speed [23].

Aiming to create polymer optical waveguides by applying different shaping methods, the present work focuses on the description of the composition-dependent physical properties of copolymers, consisting of different polyfunctional monomers, poly(methylmethacrylate) and the electron-rich molecule phenanthrene as the dopant.

## 2. Materials and Methods

**Figure 1:**
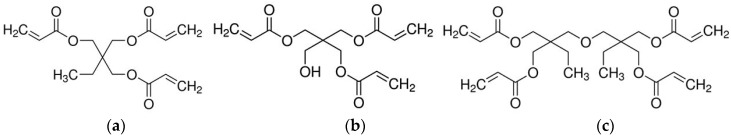
Used polyfunctional acrylate comonomers: (**a**) trimethylolpropantriacrylate (TMPTA); (**b**) pentaerythritol triacrylate (PETA); (**c**) di(trimethylolpropane) tetraacrylate (DTTA).

A commercially available premixed polymer resin (Plexit 55, Carl Roth company, Karlsruhe, Germany), consisting of poly(methylmethacrylate), (PMMA, content 30–35 wt %), solved in methylmethacrylate (MMA, 65–70 wt %) was used as the thermally or photochemically curable matrix. Three different poly-functional acrylate monomers, trimethylolpropantriacrylate (TMPTA), pentaerythritol triacrylate (PETA) and di(trimethylolpropane) tetraacrylate (DTTA, also denoted as DiTMPTA) (Figure 1), all obtained from Sigma-Aldrich (Steinheim, Germany), were used as polyfunctional acrylate crosslinker resulting in copolymers after curing. In a first series of investigations, increasing incremental amounts from 0 up to 2, 4, 8, 16 and 32 wt % of the polyfunctional acrylate monomers were added to the main Plexit 55 resin. In a second series of investigations, the new mixtures were doped with phenanthrene up to the solubility limit as a refractive index booster, as described in previous work [17,18,19,20].

All components were mixed together using a high-speed stirrer (Ultraturrax T8, IKA, Staufe, Germany) and polymerized thermally using 1 wt % dilauroylperoxide (DLP) (Sigma-Aldrich, Steinheim, Germany) at a curing temperature of 70 °C applied for 24 h. Table 1 lists the used sample series denotation in this work and the related real composition of all investigated thermally-cured mixtures. Constant amounts of thermal initiator (DLP, 1 wt %) and release agent (INT54, 1 wt %) had to be added to get 100%. Due to order of mixture fabrication (first monomer mixture, then dopant addition) the effective concentration of the polyfunctional acrylate monomers vary slightly from the denotation value.

The photochemical curing behaviour was investigated for selected mixtures applying the photoinitiator D3358 (3 wt %, TCI, Eschborn, Germany) in combination with a powerful LED light source (405 nm, LED spot 100, Dr. Hoenle, Gräfelfing, Germany). To ensure complete polymerization, 1 wt % DLP as in the other systems was also added. To avoid adhesion to the glass substrate, a release agent (INT54, E. & P. Wuertz GmbH, Bingen, Germany) was added to the curable mixtures.

The viscosity of all mixtures prior to curing was measured using a cone and plate rheometer (Malvern-Bohlin CVO50, Herrenberg, Germany, temperature range 20–60 °C, shear rate range 1–200 1/s, cone diameter 40 mm, cone inclination angle 4°, gap size 150 mm). The experimental uncertainty for viscosity values larger than 0.1 Pa s is around ±5% and ±10% for values below 0.1 Pa s. The refractive indices of uncured liquid samples and cured solid samples (mean value of at least five measurements) were characterized by an Abbe refractometer AR2008 (589 nm, 20 °C, experimental uncertainty ±0.001, Kruess, Hamburg, Germany). The optical transmittance in the 400–800 nm range of uncured liquid samples was measured applying a Cary 50 UV/Vis spectrophotometer (Varian, now Agilent, Waldbronn, Germany) using disposable UV semi-micro cuvettes (12.5 × 12.5 × 45 mm^3^ outer dimensions). The glass transition temperatures *T*_g_ of the cured systems were estimated via differential scanning calorimetry (DSC) (Netzsch, DSC204F1 Phoenix, heating rate 10 °C/min) with an uncertainty of ±2 °C. The Vickers hardness (Paar-Physica MHT10, load: 100 p, indention speed: 10 p/s, loading time: 1 s) of selected samples (mean value of five measurements, standard deviation ±2.5 units) were recorded after polymerization for suitable test specimens.

## 3. Results and Discussion

### 3.1. Shear Rate and Temperature-Dependent Viscosity of Uncured Mixtures

**Figure 2:**
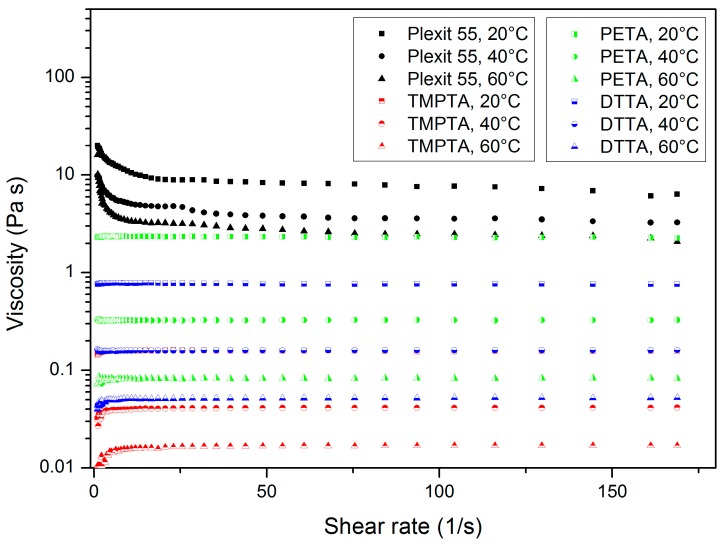
Shear rate and temperature-dependent viscosity of all investigated pure components.

The knowledge of the impact of the polyfunctional acrylate type and dopant concentration as well as the shear rate and temperature on the viscosity of the mixture is essential for further processing, micro-structuring and device fabrication applying different shaping methods. The different variants of reactive resin processing techniques possess different acceptable resin viscosities at processing or operation temperatures. Figure 2 shows for better comparison the shear rate and viscosity-dependent flow behaviour for all investigated pure components. Due to the polymer amount of around 30–35 wt % in the commercial resin, Plexit 55 possesses a pseudoplastic flow at all investigated temperatures, as described earlier [17].

The pure monomers TMPTA, PETA and DTTA show in all cases a pure Newtonian flow, in which the two non-polar monomers, TMPTA and DTTA, exhibit significant lower viscosity values than the more polar, one hydroxy-group-containing PETA. In addition, the combination of a commercial epoxy acrylate with EGDMA, the pure epoxy acrylate itself, and all derived mixtures, also showed a Newtonian flow [24].

#### 3.1.1. Trimethylolpropantriacrylate (TMPTA)-Containing Systems

**Figure 3:**
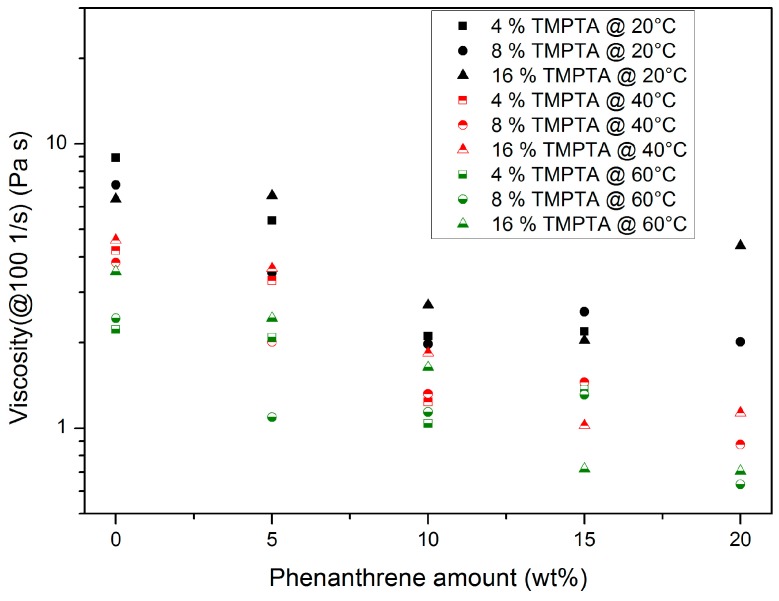
Influence of temperature, TMPTA, and phenanthrene content on the viscosity of the mixture at a fixed shear rate of 100 1/s.

The addition of increasing amounts of TMPTA turned the pseudoplastic flow into a Newtonian flow, as expected from Figure 2. Figure 3 summarizes the influence of temperature and chemical composition on the resulting mixture viscosity for a selected shear rate of 100 1/s. The main results are:

- A temperature increase causes a viscosity drop: this is a common behaviour for all organic liquids and can be described by the Andrade–Eyring relation [25].
- Increasing TMPTA amounts cause a viscosity drop due to simple mixing rules.
- Increasing phenanthrene amounts cause a viscosity drop: The given plasticizing effect of phenanthrene, solved in different reactive resins, is in agreement with other results [17,18,19,20].
- A total of 20 wt % phenanthrene exceeds the solubility limit at a TMPTA content of 4 wt %.

#### 3.1.2. Pentaerythritol Triacrylate (PETA)-Containing Systems

**Figure 4:**
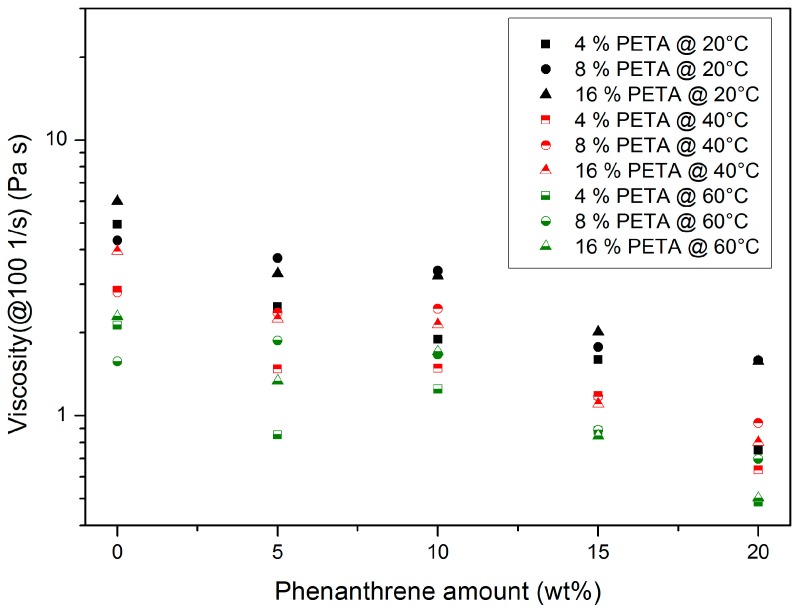
Influence of temperature, PETA, and phenanthrene content on the viscosity of the mixture at a common shear rate of 100 1/s.

The addition of increasing amounts of PETA and phenanthrene to Plexit 55 causes a comparable influence on the flow behaviour (Figure 4). A closer investigation of the viscosity data reveals a certain scattering of the viscosity data:

- At a constant PETA content, a temperature increase causes a viscosity drop.
- Increasing phenanthrene amounts cause a principal viscosity drop due to plasticizing.
- Within the investigated concentration range, phenanthrene is completely soluble up to 20 wt %.

#### 3.1.3. Di(trimethylolpropane) Tetraacrylate (DTTA)-Containing Systems

**Figure 5:**
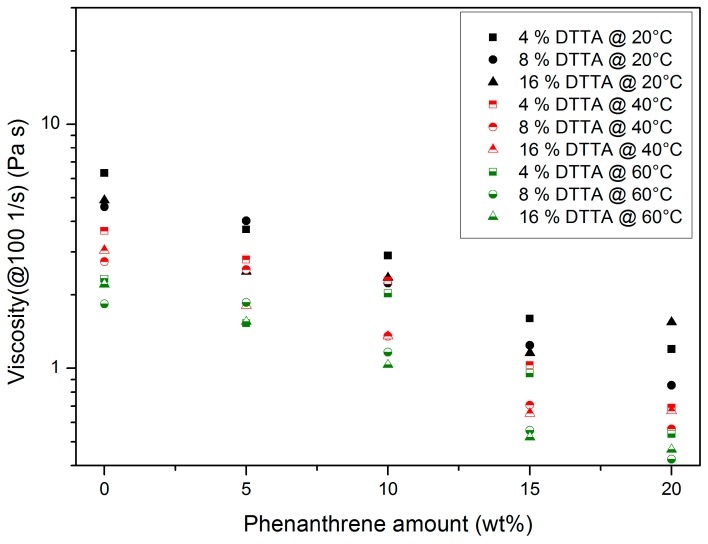
Influence of temperature, DTTA, and phenanthrene content on the viscosity of the mixture at a common shear rate of 100 1/s.

As with the other investigated systems, the addition of increasing amounts of DTTA and phenanthrene to Plexit 55 causes an almost analogous influence on the resulting mixture’s flow behaviour (Figure 5). A closer investigation of the viscosity data reveals a slight scattering of the viscosity data:

- At constant DTTA content, a temperature increase causes a viscosity drop.
- Increasing phenanthrene amounts cause a principal viscosity drop due to plasticizing.
- A total of 20 wt % phenanthrene exceeds the solubility limit at a DTTA content of 16 wt %.

In general, all three polyfunctional acrylates lower the viscosity of the Plexit 55 matrix with increasing monomer content. The addition of phenanthrene also causes a viscosity drop. In a few cases, the solubility limit of phenanthrene in the monomer mixture is lowered. The presence of the hydroxyl-functionality in PETA may prohibit a clear relationship between the composition and resulting viscosity values. The information obtained in this section enables viscosity tailoring with respect to the aspired viscosity range attributed to the selected further processing. As an example, polymer waveguide inkjet printing requires UV-curable mixtures with a viscosity below 16 mPa s at printing temperatures (40–70 °C) [11,12]. Unfortunately, none of the investigated mixtures fulfills this viscosity requirement for inkjet printing. In contrast, flexoprinting and SLA accept viscosities up to 500 mPa s, which can be achieved here.

### 3.2. Optical Properties of Uncured and Cured Systems

With respect to the fabrication of polymer waveguides using different shaping methods, the adjustment of the refractive indices of the core and cladding for waveguiding is crucial. In addition, knowledge of the optical transmittance is essential for low loss material selection.

#### 3.2.1. Refractive Index

**Figure 6:**
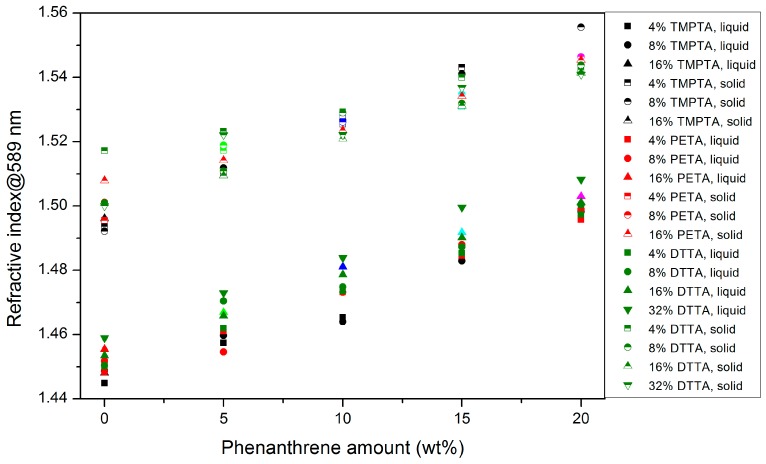
Refractive indices of all investigated mixtures prior (liquid) and after curing (solid).

The real part of the complex refractive index, typically denoted as refractivity or reactive index, is a measure of the interaction of the electromagnetic field of the incoming wave with the electronic situation in matter, especially the polarizability in the affected molecules [26]. An increase of the polarizability in organic molecules can be realized by an increase of the number of movable electrons, e.g., by the extension of delocalized π-electrons as given in the dopant phenanthrene. In addition, a further interaction—e.g., by forming charge–transfer complexes—can lead to a light absorption (yellowing) in the visible equivalent to the imaginary part of the complex refractive index. With respect to technical applications of the new materials—e.g., as waveguides—an absorption in the visible has to be strictly avoided. Figure 6 shows for all the investigated systems a change in the refractive index with mixture composition and dopant concentration. The most important correlations found were:

- Due to the lower density of the liquid monomer mixtures, the refractive index is lower in general than the related ones in the solidified state.
- All measured values are close together, the rise in the refractive index can be attributed mainly to the increasing phenanthrene moiety.
- As found in other systems, an almost linear correlation of the refractive index with the phenanthrene concentration can be derived [18,19].
- In the case of DTTA-based systems, the use of larger DTTA amounts are favorable in the liquid state.
- A clear correlation of the polyfunctional acrylate monomer structure with the refractive index values cannot be found.

#### 3.2.2. Optical Transmittance in the Visible Range

**Figure 7:**
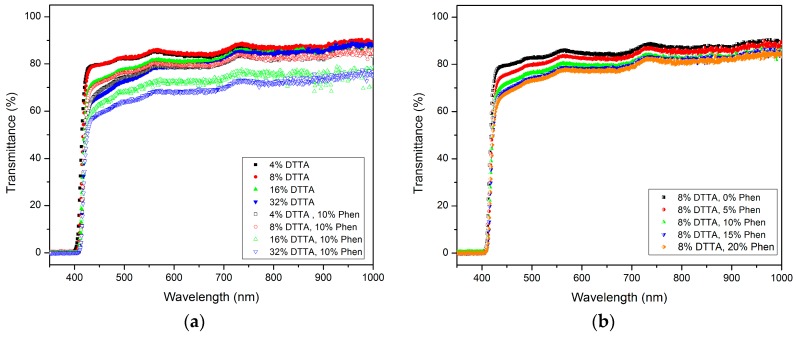
Optical transmittance of DTTA-containing mixtures: (**a**) Influence of DTTA content without and with 10% phenanthrene; (**b**) Influence of phenanthrene content at constant DTTA amount.

Exemplarily, the influence of the copolymer composition on the optical transmittance properties were investigated applying the DTTA-based mixtures. Without dopant, the influence of the increasing DTTA content was negligible; an influence was found for low amounts of DTTA in combination with a moderate phenanthrene load of 10%. Higher DTTA moieties in combination with 10% dopant resulted in some higher absorbance (Figure 7a). The combination of low amounts of DTTA with increasing phenanthrene contents up to 20% produced only a small drop in the transmittance values (Figure 7b).

Mixtures consisting of TMPTA or PETA in combination with phenanthrene showed comparable transmittance behaviour due their similar chemical structure without functional groups absorbing in the visible range.

The main result of this subsection can be summarized as follows: large refractive indices, generated with huge dopant concentrations, have the drawback of the reduced optical transmittance of the used doped copolymer. The amount of polyfunctional acrylate monomer has a certain influence on the optical properties, enabling a certain flexibility in the viscosity value for use in different shaping methods.

### 3.3. Photoinitiated Curing Behaviour

In general, the radical polymerization of acrylates suffer from the inhibiting influence of oxygen if the curing process is carried out under ambient conditions in an air atmosphere. Several negative effects can be observed during photopolymerization [27]:

- Occurrence of an induction period prior to the chemical reaction;
- Reduction of the polymerization rate and final conversion;
- Reduction of the chain length and conversion rate;
- Formation of a sticky surface appearance due to oligomer formation.

Different approaches can be applied to overcome the oxygen inhibition process [28,29,30]:

- UV-curing in an inert atmosphere;
- Preventing oxygen diffusion by protecting films or transparent foils;
- Selection of highly effective light sources like vapor pressure arc lamps;
- Use of suitable additives trapping oxygen-like triphenylphosphine [31];
- Use of monomers with huge chemical reactivity.

Especially in the latter case it is recommended to add polyfunctional acrylates to a photocurable system to enhance the cure speed, reduce the oxygen inhibition effect and enhance the resulting physical properties [23,29]. Earlier investigations applying different acrylate mixtures exhibited sticky surface properties after UV-curing in air, hence a polymerization between glass plates was necessary [32]. With respect to the verification of the positive influence of the polyfunctional acrylates on the curing behaviour [23], DTTA-containing mixtures were cured exemplarily by UV-light. Two different setups for the photopolymerization process were investigated. First, the resin compositions were placed between two glass plates and UV-cured with the exclusion of oxygen. Second, the resins were UV-cured under exposure to air. It was found that even at low concentrations of DTTA (4 wt %) both setups delivered tack-free, hard and transparent surfaces, while DTTA-free samples remained sticky at the surface. Hence, a simplified curing procedure is possible and shows the positive influence of the polyfunctional acrylate monomer. At large DTTA amounts (32 wt %) and higher phenanthrene concentrations the samples became turbid some weeks after preparation. The UV-curing of pure DTTA delivered a very brittle polymer due to thermoset formation [23]. The above results are also valid for the two other polyfunctional acrylates, PETA and TMPTA.

### 3.4. Thermomechnical Properties

One motivation for the use of polyfunctional acrylates as comonomers is their ability to form rigid polymer networks after curing. It can be expected that with a higher polyfunctional acrylate content in the resulting polymer, enhanced thermomechanical properties will be found in comparison to pure thermoplastics [23].

#### 3.4.1. Glass Transition Temperature

**Figure 8:**
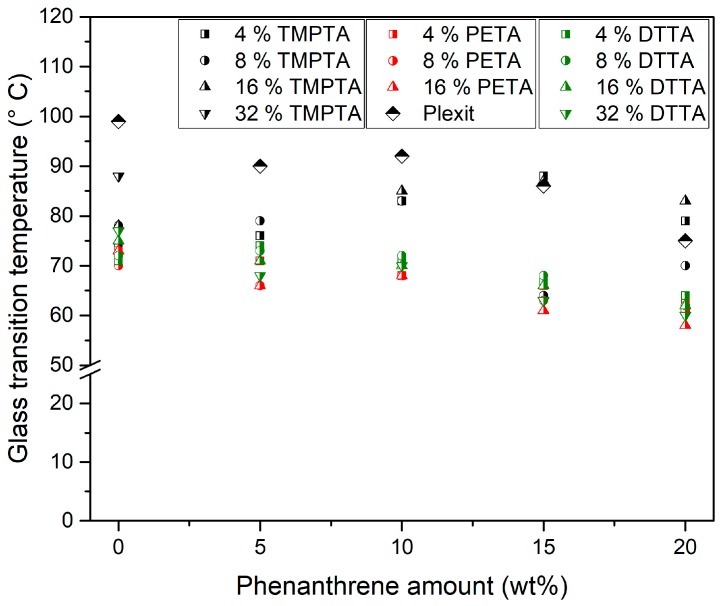
Glass transition temperatures of all investigated systems after curing.

Figure 8 shows the *T*_g_ values measured by DSC of all investigated mixtures, the data for doped Plexit without any other comonomer was added for reference. In all cases, increasing amounts of phenanthrene lowers *T*_g_ relative to the reference material (cured Plexit, i.e., PMMA) due to plasticizing, as has been reported elsewhere [17,18,19,20,21]. This is also valid for the use of a polyfunctional acrylate comonomer irrespective of whether phenanthrene is present or not. A more careful data analysis shows a certain order of the comonomers: PETA caused a pronounced *T*_g_ drop, the influence of DTTA was moderate, and TMPTA showed the smallest decrease. Higher amounts of TMPTA generated the aspired *T*_g_ elevation at higher dopant concentrations in comparison to the related Plexit/phenanthrene mixtures.

At present, inconsistent *T*_g_ values for the polymerized pure polyfunctional acrylates have been described. While [23] lists 98 °C for poly(TMPTA): 62 °C, poly(PETA): 103 °C and poly(DTTA), some other references provide different information. One group measured a glass transition around 88 °C for a doped PETA polymer [33], while in [34] no glass transition in the temperature range from −50–220 °C was found. The aspired effect of an increase in glass transition temperature, within one series, and with an increasing polyfunctional acrylate comonomer content and a constant dopant amount, can only be observed at large TMPTA amounts (Figure 8). This may be attributed to the flexible chemical structure of the comonomers allowing a more elastomeric substructure. One can assume that huge amounts of TMPTA enables the formation of a more rigid thermoset-like network due to its more compact chemical structure. This can be also deduced from the low viscosity value of the TMPTA monomer, shown in Figure 2, as an indication of low inter-molecular friction in the liquid phase due to a more compact structure.

Earlier work described that the use of rigid reactive molecules, like divinylbenzene, can increase *T*_g_ if they are used as comonomer in an unsaturated polyester–resin matrix after solidification [17]. Within an acrylate-based resin matrix, the use of 1,3-butandiol dimethacrylate (BDMA) as small difunctional molecule resulted in an observed drop of *T*_g_ with increasing amounts and a clear correlation between BDMA-content and *T*_g_ stabilization at high plasticizer content could be detected [18]. Without any crosslinker, a pronounced *T*_g_ decay with increasing phenanthrene concentration in PMMA (polymerized Plexit) and polymerized unsaturated polyester was found [19].

#### 3.4.2. Vickers Hardness

**Figure 9:**
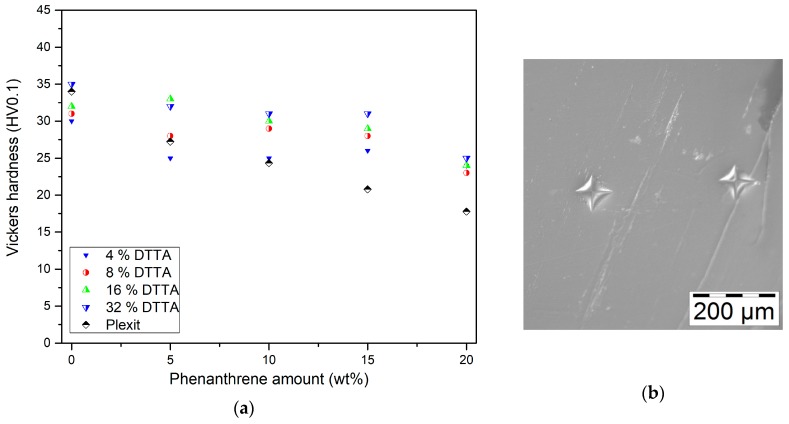
(**a**) Vickers hardness change as a function of DTTA and phenanthrene content; (**b**) Light microscope image of Vickers diamond tip indentation in a representative specimen (4% DTTA, 10% phenanthrene).

Previous work investigating phenanthrene/unsaturated polyester/divinylbenzene mixtures, described a significant increase in the Vickers hardness of cured specimen with increasing divinylbenzene content [17]. In the case of a comparable system consisting of phenanthrene/Plexit/BDMA, no remarkable hardness increase could be measured [19]. Exemplarily, the DTTA-based mixtures were investigated with respect to the influence of the comonomer and the phenanthrene content (Figure 9). In all cases, increasing dopant concentrations show a decline in hardness due to plasticizing.

In contrast to the pure Plexit based mixtures (data added to Figure 9a for better comparison, taken from [21]), the presence of DTTA at a constant phenanthrene load yields a measurable hardness increase following the DTTA concentration and enables a partial compensation of the plasticizing effect. Figure 9b shows exemplarily the diamond indentation in the specimen after hardness measurement.

## 4. Conclusions

The three different polyfunctional acrylate monomers investigated—trimethylolpropantriacrylate (TMPTA), pentaerythritol triacrylate (PETA) and di(trimethylolpropane) tetraacrylate (DTTA)—were copolymerized at different monomer ratios with a commercial MMA/PMMA-based reactive resin. With respect to the polymer waveguide fabrication, phenanthrene was added for refractive index adjustment. The shear rate and temperature-dependent viscosity was measured as a function of the uncured guest–host system, validating the applicability of different shaping methods, such as inkjet printing with a boundary condition of maximum 16 mPa s viscosity at printing temperature. After polymerization, the resulting physical properties were comprehensively characterized. The most important results of this work are:

- The resulting mixture’s viscosity can be adjusted by the addition of the polyfunctional acrylate comonomer without a pronounced impact on the optical properties.
- The presence of the polyfunctional acrylate monomer supports the photopolymerization and eliminates the negative oxygen inhibition effect.
- The polyfunctional acrylates have no significant relevant effect on the refractive index and the optical transmittance in the visible.
- Phenanthrene can be used for a pronounced increase of the refractive index.
- Increasing amounts of the polyfunctional acrylate in the copolymer can compensate the plasticizing effect of the dopant phenanthrene, enabling a higher concentration of the dopant in the guest–host system and therefore larger refractive index values.

In summary, all three investigated polyfunctional acrylates can be used as comonomers with respect to property tailoring. Considering the aspired individual target features and the scheduled shaping methods, a suitable mixture can be selected from a broad composition portfolio.

## Figures and Tables

**Table 1:** Sample series denotation and related mixture composition (in round figures to 100% without decimal place) All mixtures contain in addition 1 wt % thermal initiator DLP and release agent INT54 each.

Series Denotation	Composition [wt %]		
	Plexit55	Polyfunctional Acrylate	Phenanthrene
0 wt % series	98	0	0
	93	0	5
	88	0	10
	83	0	15
	78	0	20
4 wt % series	94	4	0
	89	4	5
	84	4	10
	80	3	15
	75	3	20
8 wt % series	90	8	0
	85	8	5
	81	7	10
	76	7	15
	72	6	20
16 wt % series	82	16	0
	78	15	5
	74	14	10
	69	14	15
	65	13	20
32 wt % series	66	32	0
	63	30	5
	59	29	10
	56	27	15
	53	25	20
